# Worldwide trends in prediabetes from 1985 to 2022: A bibliometric analysis using bibliometrix R-tool

**DOI:** 10.3389/fpubh.2023.1072521

**Published:** 2023-02-13

**Authors:** JingYi Zhao, Min Li

**Affiliations:** Guang'anmen Hospital of China Academy of Chinese Medical Sciences, Institute of Metabolic Diseases, Beijing, China

**Keywords:** prediabetes, diabetes, bibliometrics, R language, bibliometrix

## Abstract

**Background:**

Prediabetes is a widespread condition that represents the state between normal serum glucose and diabetes. Older individuals and individuals with obesity experience a higher rate of prediabetes. Prediabetes is not only a risk factor for type 2 diabetes mellitus (t2dm) but is also closely related to microvascular and macrovascular complications. Despite its importance, a bibliometric analysis of prediabetes is missing. The purpose of this study is to provide a comprehensive and visually appealing overview of prediabetes research.

**Methods:**

First, the Web of Science (WOS) database was searched to collect all articles related to prediabetes that were published from 1985 to 2022. Second, R language was used to analyze the year of publication, author, country/region, institution, keywords, and citations. Finally, network analysis was conducted using the R package bibliometrix to evaluate the hotspots and development trends of prediabetes.

**Results:**

A total of 9,714 research articles published from 1985 to 2022 were retrieved from WOS. The number of articles showed sustained growth. Rathmann W was the most prolific author with 71 articles. *Diabetes Care* was the journal that published the highest number of articles on prediabetes (234 articles), and Harvard University (290 articles) was the most active institution in this field. The United States contributed the most articles (2,962 articles), followed by China (893 articles). The top five clusters of the keyword co-appearance network were “prediabetes”, “diabetes mellitus”, “glucose”, “insulin exercise”, and “oxidative stress”. The top three clusters of the reference co-citation network were “Knowler. WC 2002”, “Tabak AG 2012”, and “Matthews DR1985”.

**Conclusions:**

The combined use of WOS and the R package bibliometrix enabled a robust bibliometric analysis of prediabetes papers, including evaluation of emerging trends, hotspots, and collaboration. This study also allowed us to validate our methodology, which can be used to better understand the field of prediabetes and promote international collaboration.

## 1. Introduction

Prediabetes is a major worldwide public health issue. Individuals with prediabetes have a high risk of progression to diabetes and elevated risks of kidney disease, cardiovascular disease, and death (1). The concept of prediabetes emerged in the late 1970s to better understand the process of diabetes (2, 3). However, it is unclear whether prediabetes should be classified as a unique pathogenic state because it is a status that lies between healthy glucose homeostasis and the pathological condition of diabetes (4). Prediabetes is a degree of impairment between euglycemia and the hyperglycemia of type 2 diabetes (5). Professional societies such as the American Diabetes Association (ADA), the World Health Organization (WHO), and the International Expert Committee (IEC) have issued definitions of prediabetes. These definitions are based on a variety of hyperglycemia-related parameters such as FBG, 2hBG, and HbA1C (6, 7).

Nevertheless, there is still no consistent definition of prediabetes, and different definitions correspond to different groups of individuals in epidemiologic studies (8). For example, large surveys of Chinese adults using all three glycemic tests (HbA1C, FBG, or 2hBG) revealed the prevalence of prediabetes, ranging from 36% in one study to as high as 50.1% in another (9). Previous literature also suggested that, for individuals over 40 years of age or with a higher risk of diabetes, FBG and/or HbA1C were more effective (10). For individuals with prediabetes, pharmacological and lifestyle changes could reduce cardiovascular risk and cost-effectively prevent diabetes (11), and restoring normoglycemia can produce long-lasting remission (10). Hence, the National Institute for Health and Care Excellence (NICE) suggested that individuals with prediabetes should initially undergo lifestyle intervention in the form of intensive group education programs (12). However, the effectiveness of these interventions relies on a consistent and accurate definition of prediabetes.

Insulin resistance, B-cell dysfunction, increased lipolysis, inflammation, poor incretin response, and hepatic glucose overproduction are all pathophysiologic abnormalities that underlie prediabetes (13). Obesity-related metabolic abnormalities increase the risk of macrovascular and microvascular problems by impairing endothelial vasodilators and fibrinolytic activity. Additionally, prediabetes has been linked to an increased risk of cancer and dementia (14, 15).

Bibliometric analysis has evolved into the most effective tool for investigating detailed research trends in a research field over time. It objectively presents research contributions related to particular scientific fields from different countries, institutions, journals, and authors through statistical analysis and forecasts future directions or hotspots (16). It is important to note that hotspots flag emerging problems in a specific field that have not been resolved and are of great concern to global academics, and future research directions forecast research that must be undertaken urgently and that will have a significant impact in the future. Furthermore, bibliometric analysis has played a significant role in the development of policy and clinical guidelines for a variety of diseases. However, to date, no bibliometric analysis of prediabetes has been conducted, and even less attention has been given to the prediction of research hotspots.

In this research, we retrieved prediabetes-related articles from the Web of Science (WOS) database and used bibliometric analysis tools to examine the literature characteristics and research hotspots. The Web of Science (WOS) is the most comprehensive and authoritative citation database in which peer review is a requirement in the journal evaluation process for inclusion. Therefore, we chose WOS in this study. The goal of this study is to provide a comprehensive and visually appealing overview of prediabetes studies and to lay a robust foundation for future research.

## 2. Methods

The Core Collection of WOS was searched to obtain relevant literature. The search strategy was as follows: TS = Prediabet^*^ AND PY = (1985–2022). The search was performed on 17 August 2022. Only articles and reviews were included in the analysis. Two researchers independently retrieved and downloaded the literature. After data confirmation and standardization, the online literature was exported to plain text format, including full documents and cited references. The data were then imported into R for analysis.

We used the R package bibliometrix to clean, analyze, and visualize the literature data. Bibliometrix was created by Massimo Aria and Corrado Cuccurullo and built in R, a programming language for statistical computing and graphics (17). It contains all the necessary instruments to pursue a complete bibliometric analysis, following the Science Mapping Workflow. It is a powerful tool because it makes bibliometric analysis more sophisticated and replicable.

## 3. Results

We used the R package bibliometrix to analyze the quantity of prediabetes literature, and publications of different journals, authors, countries, and institutions. We used keyword analysis, themes, and theme evolution to understand the main research areas of prediabetes articles. We also used citation analysis to explore the logical relationships between the literature and a collaboration network to show the collaboration between countries, institutions, and authors in this field.

### 3.1. Annual literature quantity and growth forecast

**Figure 1:**
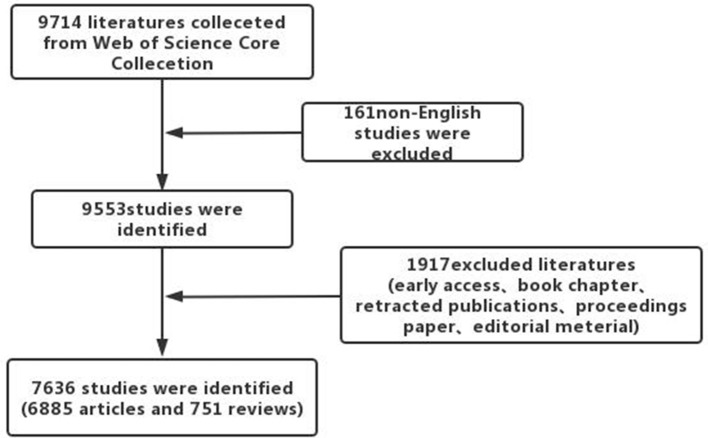
Bibliometric analysis of prediabetes presented in the workflow.

**Figure 2:**
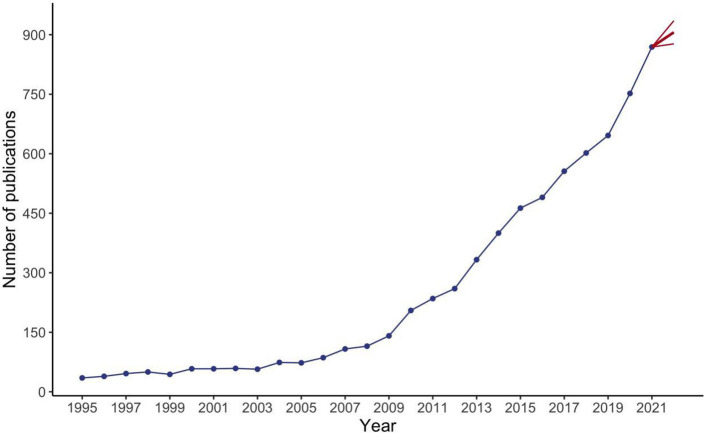
Growth trend and prediction of prediabetes.

A total of 9,714 papers were collected from the WOS (see the workflow in Figure 1). We excluded 161 non-English papers and other 1,917 papers, including early access publications, book chapters, retracted publications, proceedings papers, and editorial materials. Thereafter, 7,636 publications remained for analysis. The first article in the field of prediabetes was written by A R Dian and published in the journal “Diabetologia” (18). As shown in Figure 2, the number of articles each year has exhibited a sustained growth trend since 2005 and reached 876 in 2021. Furthermore, we ran a polynomial regression model to predict how many articles will be published in 2022. The predicted number of articles in 2022 was 906 with a 95% confidence interval of 876 to 935.

**Figure 3:**
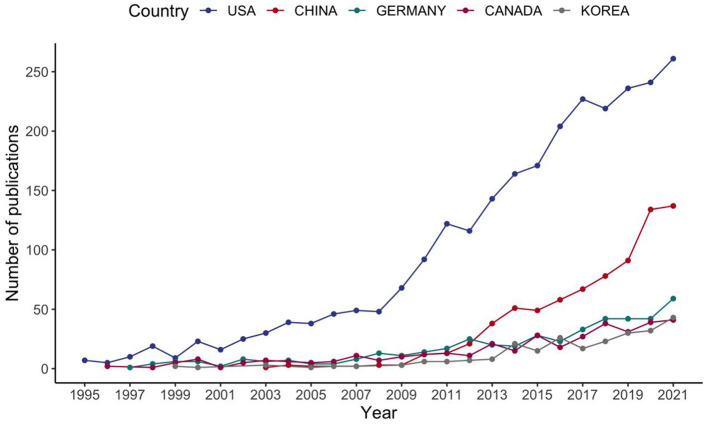
Number of publications in different countries and their growth trends.

The above pattern suggests that prediabetes is an emerging field. As shown in Figure 3, the number of articles by country also demonstrated an increasing growth trend. The United States published the most articles, followed by China, Germany, Canada, and South Korea. However, while studies on prediabetes have increased significantly over the past few decades, it is still a relatively new and promising area of research. China, India, Pakistan, and the United States (US) are the countries with the largest numbers of patients with diabetes aged 20–79 years in 2021. The US and China have the highest interest in the area of prediabetes because of the high prevalence of diabetes and the high economic level in these countries. India and Pakistan ranked only 10th and 44th in the prediabetes field in terms of the number of publications, which may be related to the investment in research and the emphasis on diabetes prevention.

### 3.2. Distribution of literature

We then analyzed the distribution of authors, journals, and institutions of the literature. More than 34,914 authors contributed to the 7,636 prediabetes-related studies published in the WOS. Among the 20 most-productive authors, Rathmann W had the most publications (71 articles), followed by Peters A (58 articles). Haring HU and Meisinger C were tied for third place (47 articles each) (Supplementary material 1).

The articles on prediabetes were published in more than 1,549 journals. *Diabetes Care* published 234 articles, which accounted for 3.02% of all articles, followed by “PLoS ONE” (193 articles*)*, “Diabetes Research and Clinical Practice” (187 articles), “Diabetologia” (186 articles), and “Diabetes” (167 articles) (Supplementary material 2). The impact factor (IF) is a widely used indicator measuring the academic impact of a journal and the quality of its publications. Among the top five journals, *Diabetes Care* had the highest IF, reaching 17.152 in 2022; the IF of the other four journals, i.e., *Diabetologia, Diabetes, Diabetes Research, Clinical Practice*, and *PLoS ONE* were 10.12, 9.46, 5.60, and 3.75, respectively. A majority of the prediabetes-related articles published in these journals were of high quality and worth further analysis.

According to the retrieval results of the WOS database, the authors were affiliated with 139 countries/regions. The United States was the country with the highest number of publications (2,962 articles), followed by China (893 articles), Germany (471 articles), England (446 articles), and Canada (398 articles) (Supplementary material 3). Notably, the literature on prediabetes in China has shown rapid growth in the last decade.

In terms of affiliations, there were 7,834 institutes involved in the field of prediabetes. Harvard University ranked first with 290 articles on prediabetes, followed by the University of California (269 articles), the U.S. Department of Veterans Affairs (194 articles), Veterans Health Administration (192 articles), and the University of Texas system (191 articles). Eight of the top 10 institutions were located in the US (Supplementary material 4). There is no doubt that the US has maintained its lead in the field of prediabetes. Shanghai Jiao Tong University was the top Chinese institution in terms of the number of articles on prediabetes and ranked 22nd overall.

### 3.3. Keywords analysis

Keywords are brief phrases used in indexing or classifying to describe the topic of an article accurately and concisely. Through keyword analysis, we can gain a general understanding of the themes and features of publications (19). Co-occurrence analysis assumes that keywords in the same documents are strongly related to the conceptual space of the research area. Clustering the keyword co-occurrence network provides a method to identify the subfields of a research area (20). We used author keywords in the following analysis and built a network with 8,960 nodes and 51,101 links.

Research frontiers can be identified by examining the frequency and centrality of keywords (21). The top 20 most common keywords are shown in Table 1. “Prediabetes” was the most commonly used keyword in the literature, followed by “diabetes”, “type 2 diabetes”, “diabetes mellitus”, “insulin resistance”, “obesity”, “metabolic syndrome”, “HBA1C”, “IGT”, “IFG”, and “Insulin”.

**Table 1:** Top 20 keywords of prediabetes.

**Ranking**	**Counts**	**Centrality**	**Keywords**
1	2,284	0.387	Prediabetes
2	1,497	0.247	Diabetes
3	903	0.118	Type 2 diabetes
4	526	0.048	Insulin resistance
5	478	0.043	Obesity
6	256	0.013	Metabolic syndrome
7	246	0.011	HBA1C
8	226	0.012	IGT
9	179	0.007	IFG
10	151	0.015	Insulin
11	150	0.018	Type 1 diabetes
12	143	0.008	Inflammation
13	137	0.005	Cardiovascular disease
14	135	0.007	Prevention
15	134	0.004	Risk factors
16	127	0.005	Hyperglycemia
17	106	0.004	Hypertension
18	106	0.005	Secretion of insulin
19	106	0.007	Metformin
20	104	0.005	Epidemiology

The top 100 keywords can be classified into five groups: prediabetes-related diseases, diagnostic criteria, risk factors, intervention modalities, and pathological mechanisms (Supplementary material 5). Diseases frequently addressed in the literature on prediabetes are “obesity”, “metabolic syndrome”, and “cardiovascular disease”. Among them, “type 2 diabetes” appeared more frequently as a keyword than “type 1 diabetes”. “Insulin resistance”, “inflammation”, and “sensitivity to insulin” were popular pathological mechanisms in prediabetes. The discussions of the diagnostic criteria in order of frequency were “HBA1C”, “OGTT”, and “FPG”. Physiological indicators such as “BMI”, “blood pressure”, and “waist circumference” caused relatively high concern; “metformin”, “exercise”, and “physical activity” were the most frequently studied interventions in the field of prediabetes.

### 3.4. Cluster analysis of keywords: Cooccurrence

**Figure 4:**
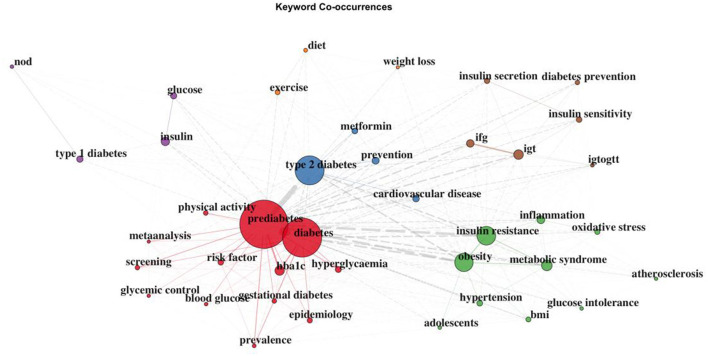
Co-occurrence network of the top 400 keywords.

In Figure 4, we demonstrate the co-occurrence network of the top 400 most frequent keywords. They are clustered into five categories: “prediabetes”, “type 2 diabetes”, “insulin resistance”, “exercise”, and “insulin”.

Keywords in the same cluster were presented by the same color, and they were clustered together because they often appeared together in the same article. The purple cluster contained four major keywords: type 1 diabetes, nod, glucose, and insulin. “Nod” is commonly used for modeling “type 1 diabetes”, and loss of “insulin” secretion is a key mechanism for the progression of prediabetes to “type 1 diabetes”. The red cluster gathered the most articles. It represented some basic questions about prediabetes such as prevalence, risk factors, and screening. The green cluster was related to the study of prediabetes mechanisms, such as insulin resistance, inflammation, and oxidative stress, and the clustering of prediabetes-related diseases, such as hypertension, metabolic syndrome, and atherosclerosis. The brown cluster contained some common measures of prediabetes, such as IFG, IGT, OGTT, insulin secretion, and insulin sensitivity. The blue cluster contained common types of prediabetes, such as IFG and IGT, and combined the most directly related diseases together, such as type 2 diabetes and cardiovascular disease. The orange cluster mainly reflected lifestyle interventions for prediabetes such as diet, exercise, and weight loss.

**Figure 5:**
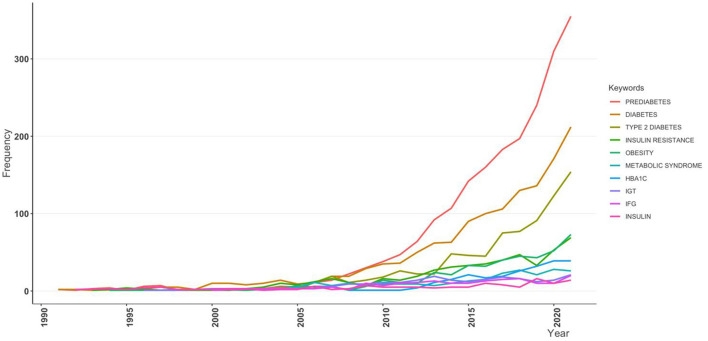
Growth trend of the top 10 keywords.

**Figure 6:**
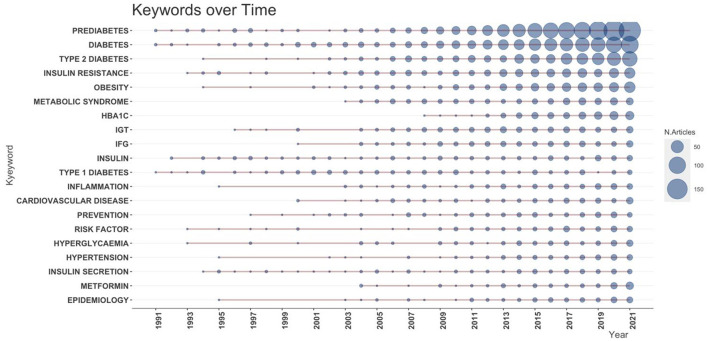
Production of the top 20 keywords over time.

When taking the time dimension into the analysis, Figures 5, 6 show that *prediabetes, diabetes*, and *type 2 diabetes* were the top three keywords in almost all periods, demonstrating the dominance of these three keywords. Between 2005 and 2007, *obesity* ranked in the top three one time, and then, it was surpassed by other keywords. Another interesting pattern is that the use of *insulin resistance* as a keyword increased very quickly since 2010 and ranked fourth in 2020, which may suggest that it is an emerging research direction.

### 3.5. Themes and thematic evolution

**Figure 7:**
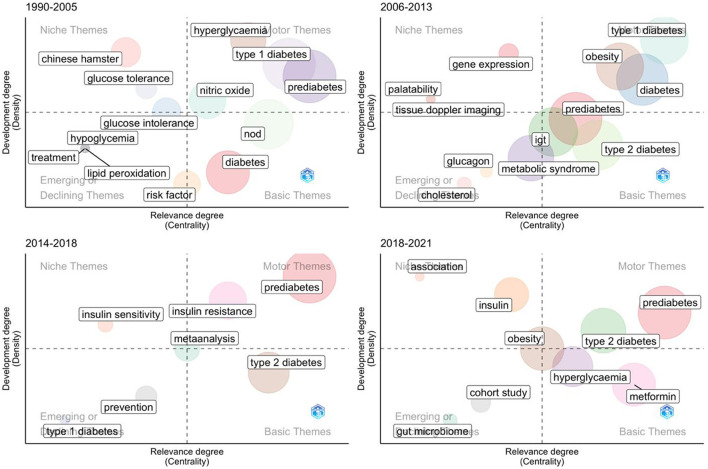
Strategic diagram for four periods.

The themes included the title, abstract and keywords, and features by conceptualization and normalization. To investigate the dynamic pattern of the research theme over time, we mapped all clusters into a strategic diagram using two metrics: centrality and density; the degree of interaction between clusters is referred to as centrality, and the degree of internal cohesion is referred to as density (22). The strategic diagram has four quadrants (Figure 7) and the themes can be categorized into four groups: (a) motor themes in the upper-right quadrant which are well-developed and relevant for the research field; (b) basic and transversal themes in the lower-right quadrant which are considered relevant for the research field, but not fully developed; (c) emerging or declining themes in the lower left quadrant which are poorly or marginally developed, and (d) highly developed and isolated themes in the upper-left quadrant which are well-developed but not relevant for the research area. The size of a given cluster is dependent on the number of keywords it contains, and the label cluster conforms to the cluster's most frequently used word. The Walktrap algorithm was used to cluster the data in this study (23).

As shown in Figure 7, the total period was split into four sub-periods: 1990–2005, 2006–2013, 2014–2018, and 2019–2022. The reason for keeping the last period so short, at only 4 years, was to gain a better understanding of current trends.

In the first period (1990–2005), the fully developed themes were related to “type 1 diabetes”, “prediabetes”, “hyperglycemia”, and “nitric oxide”. At that time, scientists believed that the early prediabetic process may be a suitable target for immunomodulation aimed at delaying or preventing progression to type 1 diabetes. The niche themes included Chinese hamster and glucose tolerance, which were not developed into moto themes in the following period. Diabetes was among the basic themes.

In the second period (2006–2013), “type 1 diabetes” remained a fully developed theme. Taken together, the period from 1990 to 2013 had much research focused on type 1 diabetes. However, after 2014, research on “type 2 diabetes” emerged and finally became the motor theme in the last 4 years (2019-2022). Notably, “obesity” and “diabetes” were the other two developed themes in the second period. “Prediabetes” was still a basic theme, despite its larger density. There were also several new niche themes, such as gene expression, palatability, and tissue Doppler imaging.

From 2014 to 2018, the theme of “type 1 diabetes” decreased while the density and centrality of “type 2 diabetes” increased. “Prediabetes” became a new motor theme, together with “insulin resistance”. Moreover, “meta-analysis” emerged as a new theme with moderate centrality and density. After a period of development, scholars reviewed and examined the existing findings of prediabetes.

In the most recent period from 2019 to 2022, “prediabetes” remained a motor theme, and “type 2 diabetes” finally joined the motor quadrant. The research on “insulin” merged into a single cluster in this period. The basic theme quadrant included two new clusters: “metformin” and “hyperglycemia”. Moreover, “hyperglycemia” was a motor theme during the first period.

In summary, the most solid theme identified in the thematic evolution was “prediabetes”, which is also the most frequent keyword over time. We also found that the research interest shifted from “type 1 diabetes” to “type 2 diabetes”. “Obesity” and “insulin” topics were also relatively solid. However, the identified niche themes were basically different for different periods. This may suggest that the research interests changed rapidly over time.

### 3.6. References analysis

Table 2 presents the top 20 most highly cited references. Eleven of these articles were written in the United States, followed by China (three articles). The epidemiology of prediabetes was the subject of one-fourth of the 20 most frequently cited articles. The most frequently cited article “Prevalence of diabetes among men and women in China” (24), was published by Yang WY in the *New England Journal of Medicine* in 2010.

**Table 2:** Top 20 most highly cited articles of prediabetes.

**Rank**	**Citations**	**Citations/year**	**Centrality**	**Year**	**First author**	**Journal**
1	2,315	178.1	5.01E-03	2010	Yang WY	New England Journal of Medicine
2	2,042	204.2	3.18E-03	2013	Xu Y	JAMA
3	1,653	71.9	8.06E-04	2000	Salomon B	Immunity
4	1,329	120.8	5.10E-02	2012	Tabak AG	Lancet
5	1,288	92	1.14E-04	2009	Scheer FAJL	Proceedings of the National Academy of Sciences of United States of America
6	1,276	116	1.01E-02	2012	Chen L	Nature Reviews Endocrinology
7	1,151	143.9	4.38E-03	2015	Menke A	JAMA
8	1,127	140.9	3.02E-04	2015	Zeevi D	Cell
9	1,097	99.7	3.74E-06	2012	Booth FW	Comprehensive Physiology
10	1,037	207.4	2.72E-04	2018	Saklayen MG	Current Hypertension Reports
11	981	163.5	3.08E-03	2017	Wang LM	JAMA
12	947	30.5	2.01E-03	1992	Martin BC	Lancet
13	944	37.8	3.11E-04	1998	Shimabukuro M	Proceedings of the National Academy of Sciences of United States of America
14	917	34	1.48E-05	1996	Yamagata K	Nature
15	868	31	8.33E-04	1995	Unger RH	Diabetes
16	863	107.9	6.79E-04	2015	Pi-Sunyer X	New England Journal of Medicine
17	856	25.9	2.14E-03	1990	Haffner SM	JAMA
18	854	47.4	5.46E-06	2005	Krentz AJ	Drugs
19	689	28.7	2.86E-05	1999	Perseghin G	Diabetes
20	687	49.1	4.75E-04	2009	Eizirik DL	Nature Reviews Endocrinology

The literature type was assessed by reading the title and abstract of the top 100 articles. Table 3 shows the literature types of the 100 most frequently cited articles in the last 3 years. Cardio-cerebrovascular complications and gut microbiota-related studies are the two research directions that have been highly cited in the past 3 years, accounting for 20% of the 100 most frequently cited articles. Clinical trials and randomized controlled trials are the most common types of literature in the field of prediabetes, accounting for 20% of the 100 most frequently cited articles.

**Table 3:** The research types of the 100 most highly cited articles (2020–2022).

**Type of research**	**Number**	**Percentage**
Cardio-cerebrovascular complications	13	13%
Clinical Trial	12	12%
Randomized controlled trial	11	11%
Epidemiology	10	10%
Review	10	10%
Experimental Research	8	8%
Gut Microbiota-related studies	7	7%
Meta-analysis	6	6%
Pathophysiology	5	5%
Diagnostic techniques	4	4%
Cohort study	4	4%
Medical care	4	4%
Guidelines	3	3%
Lifestyle intervention	2	2%

### 3.7. Cluster analysis of references: Co-citations

**Figure 8:**
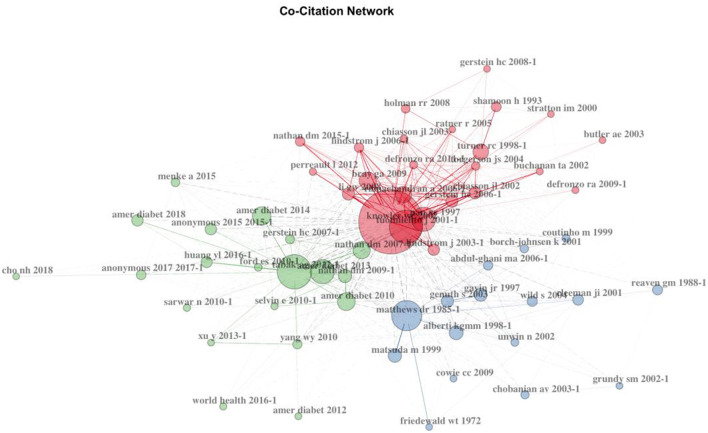
Co-citation network.

To better understand the relationship among the references, we clustered them based on the co-citation network using bibliometrix. As shown in Figure 8, three groups were obtained: “Knowler. Wc 2002”, “Tabak ag 2012”, and “Matthews Dr1985-1”. The most highly cited article in the red cluster, “Reduction in the Incidence of Type 2 Diabetes with Lifestyle Intervention or Metformin” (25), was published in 2002 in the *New England Journal of Medicine*. The most highly cited article in the green cluster, “Prediabetes: a high-risk state for diabetes development” (26), was published in 2012 in *Lancet*. The most highly cited article in the blue cluster, “Homeostasis model assessment: insulin resistance and beta-cell function from fasting plasma glucose and insulin concentrations in man” (27), was published in 1985 in *Diabetologia*.

Citations featured in the red cluster had the highest number of total citations, and their prediabetes related articles were especially significant in the first period. Most of them were published in high-impact journals such as the *Lancet* and *the New England Journal of Medicine*. The majority of the studies were long-term follow-up studies to investigate the prevalence of diabetes and related diseases and the impact of lifestyle interventions. Citations featured in the blue cluster were also less consistent in terms of their topics. They covered the longest time span (1972–2009). Most of the cited literature focuses on the detection, evaluation, and treatment of blood glucose, blood pressure, and blood lipids. Most were published in *Diabetes Care*. The green cluster cited many important prediabetes guidelines and expert consensus. These studies were relatively new, concentrated in the third period, and were mostly published in *Diabetes Care*.

### 3.8. Collaboration network

We clustered the countries and authors based on their collaboration network using bibliometrix. The nodes in the collaboration network were authors or countries, and the links represented co-authorship.

**Figure 9:**
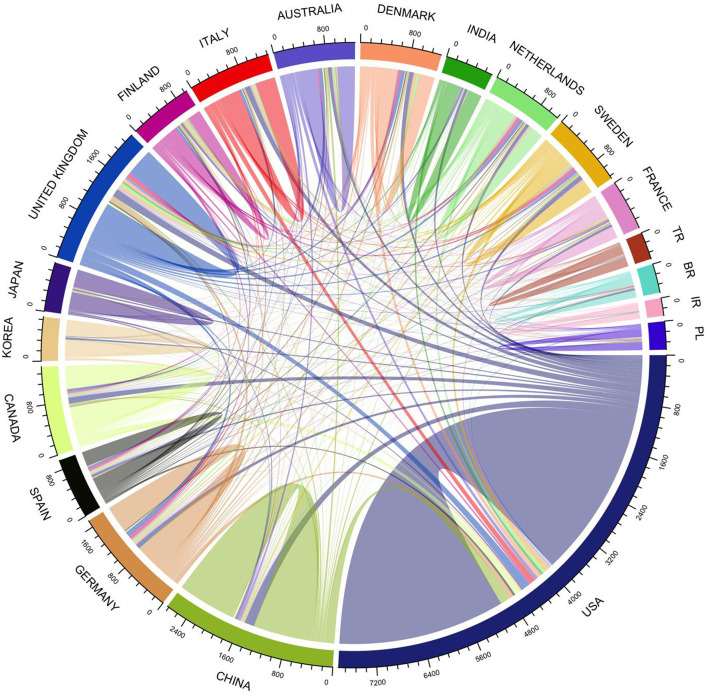
Country collaboration network.

The collaboration network between countries can be seen in Figure 9. The US was the country with the most international collaboration in the field, followed by the United Kingdom, Germany, Denmark, and Australia. It is worth noting that, while China was the second most active country in terms of the number of articles, it ranked sixth in international collaboration. In terms of the frequency of collaboration, the top five country pairs were all between the United States, China, the UK, Italy, Canada, and Germany.

**Figure 10:**
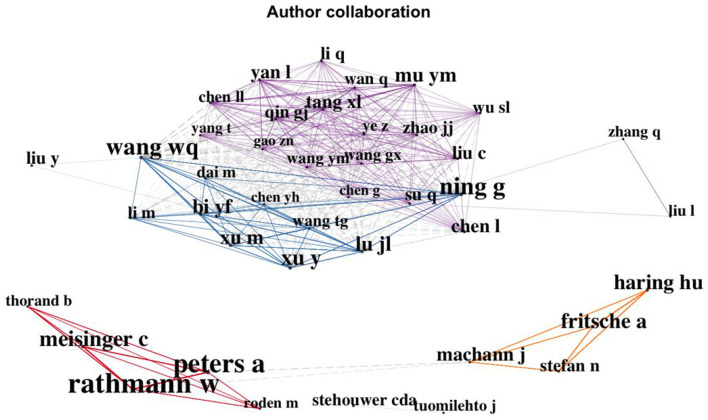
Author collaboration network.

The authors' collaboration network (Figure 10) was mapped into four clusters. Each color in this network represents a single cluster or a group of collaborating authors. Figure 10 shows that the collaborators were mostly from the same country or region. Most of the collaborative studies were large clinical trials, cohort studies, or randomized controlled studies of diabetes, prediabetes, and related diseases. These studies require collaboration between research institutions. Authors clustered in the red and orange groups were from Germany. However, the authors of the orange cluster were all from the University of Tübingen. The authors clustered in the blue and purple clusters were from China; the authors of the blue cluster were all from Shanghai Jiao Tong University.

**Figure 11:**
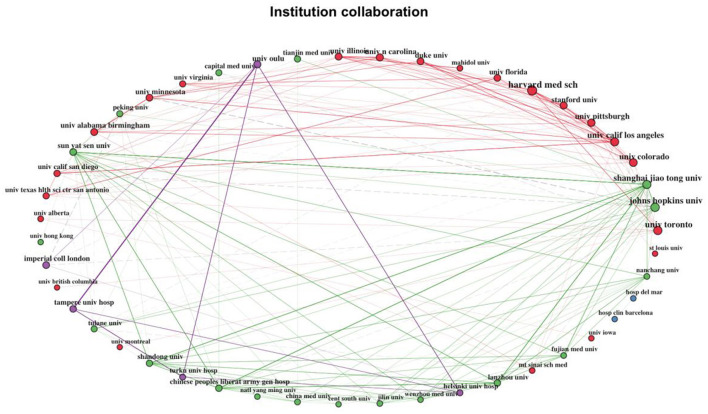
Institution collaboration network.

There were four clusters in the institution collaboration network (Figure 11). In the purple cluster, all but Imperial College London were Finnish universities. In the green cluster, all institutions were Chinese universities and hospitals, except for Tulane University and Johns Hopkins University. The US and Canadian universities comprised the red cluster. Mahidol University in Thailand also belonged to the red cluster. Finally, two institutions from Spain formed the blue cluster.

## 4. Discussion

The general term “prediabetes” refers to the stage between normal glucose tolerance and T2DM. It is generally recognized that individuals with prediabetes are at a high risk of developing T2DM (28). The bibliometric analysis is a useful tool for mining information about a research field. Through a bibliometric analysis, researchers can quickly capture the characteristics and hot spots of the literature in a specific field (19). Therefore, a comprehensive understanding of prediabetes could be obtained by using the bibliometric analysis method, which contributes to subsequent research and clinical treatment.

However, there are differing viewpoints regarding the necessity of and criteria for the diagnosis and intervention of prediabetes. Institutions such as the WHO, the National Institute for Health and Care Excellence (NICE), the European Association of the Study of Diabetes (EASD), and the International Diabetes Federation (IDF) do not use or emphasize the term prediabetes, and they normally advise treatment only when blood sugar levels approach those of diabetes. The ADA and the Centers for Disease Control and Prevention (CDC) fund much of the nation's research and programs on diabetes prevention. The ADA criteria and the WHO criteria are currently the two most commonly used criteria for prediabetes. Different from the WHO standard, in the ADA standard, the lower FPG cutoff point value of IFG was reduced to 5.6 mmol/L and included glycosylated hemoglobin (HbA1C) from 5.7 to 6.4% as one of the diagnostic criteria for prediabetes. The lower cutoff value defined by the ADA guidelines led to much higher prevalence rates compared with those defined by the WHO guidelines. In a cohort of 1,547 American adults without diabetes, changing the lower IFG threshold from 110 to 100 mg/dL resulted in an increase in prediabetes prevalence from 19.8 to 34.6% (29).

Currently, the frequency and rate of prediabetes progression to diabetes are unclear. Whether prediabetes itself causes harm is not clear, particularly when a person's average glucose levels are at the low end of the test result spectrum. The CDC data show a progression from prediabetes to diabetes at a rate of <2% per year or <10% in 5 years. The Cochrane Library in London showed that up to 59% of prediabetes patients returned to normal glycemic values over 1–11 years with no treatment whatsoever. Therefore, the diagnosis and treatment of prediabetes is not only a medical problem but also a social and economic problem. More research is still needed to determine a suitable definition and other tipping points in identifying the risk of progression to diabetes and other complications^1^

This study examined the progression of prediabetes-related research during the last 37 years. Since 2005, the number of articles on prediabetes has been increasing steadily. With the improvement of living standards and unhealthy behaviors such as physical inactivity, the incidence of prediabetes has increased significantly (30). The booming literature on prediabetes reflects the growing awareness of the importance of detecting and treating prediabetes. Over 98% of the articles were written in English. The majority of the articles were published by corresponding authors from the United States, China, Germany, Canada, and South Korea. These countries face a high incidence of diabetes and emphasize disease prevention (31). The majority of prediabetes relationships are similarly based in the United States, which is consistent with the country's substantial contribution to this academic subject, indicating that collaborations with other countries/territories should be strengthened. As a country with the second-highest number of prediabetes articles, China only ranks sixth in international collaboration. Therefore, as the country with the highest incidence of diabetes, China should strengthen international collaboration in the future to improve the ability to diagnose, prevent, and treat prediabetes.

In terms of authorship, the 20 most prolific authors have written 786 articles, accounting for 10.3% of all papers. They have made significant contributions to the development and progression of prediabetes research. Rathmann W was the most prolific author (71 articles) followed by Peters A (58 articles). The journal *Diabetes Care* published most of the literature relevant to prediabetes among the top 20 medical journals. It also had the highest IF, which reached 17.152 in 2021, demonstrating its superiority in quantity and quality. Furthermore, eight of the top 20 journals were American journals, reflecting the US's considerable interest and leadership in this field. Eight of the top 10 institutions were from the US, and Harvard University ranked first. The collaborators tended to come from the same country or region. China and Germany were the two countries with the highest concentration of collaboration networks, especially Shanghai Jiao Tong University in China and the University of Tubingen in Germany.

According to the 2021 worldwide diabetes atlas issued by the International Diabetes Federation (IDF), China, India, and Pakistan had the highest number of people with diabetes among the 20- to 79-year-old population in 2021(31). The highest diabetes-related health expenditure was observed in the United States (USD $379.5 billion), followed by China and Brazil (USD $165.3 billion and USD $42.9 billion, respectively). Both China and the United States attach great importance to diabetes prevention. The idea of “preventive treatment of disease” has existed in China since ancient times and a series of guidelines, such as the “Guideline for the prevention and treatment of type 2 diabetes mellitus in China (2020 edition)”(32), which has been published. The Diabetes Prevention Program (MDPP) had already been launched in 25 centers in the United States (33). As shown in this study, the United States and China have published the most articles in the prediabetes field. Low- and middle-income countries have higher numbers of people with diabetes and higher growth rates of diabetes prevalence. However, we found that their attention to prediabetes is low. In terms of economic development, although large-scale screening and education for prediabetes also require high financial investment, it may reduce the incidence of diabetes and the economic burden of diabetes and diabetes complications in the long run. India and Pakistan rank third and fourth in the number of patients with diabetes, respectively, but rank 10th and 44th in the area of prediabetes publications. India's research and development (R&D) intensity was only 0.66% in 2018 (34), much lower than that of 2.14% in China^2^. The disparity is a result of less research investment, lower diabetes-related health expenditures, and insufficient attention to diabetes prevention (35). On the one hand, the national annual cost associated with the diagnosis of diabetes is USD $327.2 billion and that for prediabetes is $43.4 billion. The economic burden of diagnosed diabetes may be reduced by intervening in prediabetes (36). On the other hand, screening and education for prediabetes may also pose a financial burden. The ADA, CDC, and other organizations have already spent billions on research, education, and health improvement programs. To date, no studies have been undertaken to calculate whether the investment in diagnosing and treating prediabetes can reduce the cost of diabetes treatment due to failure to intervene early.

A detailed reading of the literature in the field of prediabetes over the past 3 years revealed that 13% of the articles were related to cardiovascular risk (37). “Insulin resistance”, “inflammation”, and “sensitivity to insulin” are common mechanisms in the field of prediabetes (38–40). Research related to gut microbes is an emerging hot topic in the field of prediabetes over the past 3 years (41). Epidemiological studies accounted for 10% of prediabetes studies. Much attention has been given to the prevalence of prediabetes. However, there is no consensus on the definition of prediabetes. The complexity of defining prediabetes makes it challenging to obtain profiles of relative prediabetes prevalence from the literature (42). At least five different definitions have been endorsed by different clinical organizations and guidelines. Comparisons of incidence rates between countries will be meaningful only if diagnostic criteria are standardized.

The classification of prediabetes is mainly based on plasma glucose, which is divided into impaired fasting glucose (IFG) with elevated FPG and normal OGTT and into impaired glucose tolerance (IGT) with elevated OGTT and normal FPG. In addition, there are classification of IFG + IGT as well as classification with elevated glycated hemoglobin (HbA1C) (43). In the ranking of keywords in articles related to prediabetes, IGT appeared as a keyword in 205 prediabetes articles and ranked 9th. IFG appeared as a keyword in 165 articles and ranked 11th. HBA1c appeared as a keyword in 135 articles and ranked 14th. It can be seen that the type of prediabetes has received much attention in the field of prediabetes. We are not sure about the effectiveness of different types of prediabetes for the assessment and prevention of diabetes conversion. Further research is needed to explore blood glucose (FPG, OGTT) and HbA1C in identifying the risk of progression to diabetes and whether there are other tipping points. Further research is needed to determine which of the current definitions of prediabetes has the highest ability to discriminate between individuals who transition to diabetes and those that do not and to see how their performance varies with age, sex, and geographic location.

Clinical trials and randomized controlled trials accounted for 23% of the prediabetes literature in the last 3 years. This shows the strong need to develop an appropriate prediabetes intervention. To date, no drugs have been approved specifically for prediabetes, meaning that doctors are limited to prescribing diabetes drugs or other medications “off label” to treat the condition. Metformin is the most commonly used drug (44). However, metformin is not always prescribed for prediabetes, even if a patient meets the prediabetes criteria. Only people who are at a higher risk for developing type 2 diabetes or who have more risk factors may benefit from metformin therapy. Risk factors include having a higher body mass index (BMI) and prior gestational diabetes (45). Exercise, physical activity, and diet are common lifestyle interventions (46–48). With early detection and simple lifestyle changes (such as diet and exercise), prediabetes is often reversible (49–51). However, 38% of the lifestyle treatment group failed to maintain the strict regimen after only 6 months. More studies are needed to determine the best method and timing for intervention in prediabetes.

This study explores research trends and hotspots of prediabetes, which is useful to many researchers. On the one hand, researchers can use the research trend to prevent certain obsolete research on specific themes, reduce the repetitive effort in research initiatives, and reduce project funding waste. On the other hand, depending on research hotspots, researchers can optimize and improve their study design, making prediabetes research more novel and realistic. This study also presents a timeline of the changes in prediabetes research. It lays the groundwork for precise prediabetes prevention and treatment and provides a necessary reference value for the formulation of prediabetes guidelines and the adjustment of medical insurance policies. Ultimately, more individuals will benefit from lessening the medical load as well as the economic costs associated with prediabetes prevention and treatment around the world.

However, the limitations of this study must be mentioned. First, this study only examined publications in English, which might have led to bias in the study outcomes. Second, we only retrieved data from the WOS database and did not search additional databases or preprint articles for information, resulting in inadequate literature collection. Finally, while bibliometric analysis is a strong tool for revealing precise study trends, it provides little information about research content, such as methods or results. More review studies are needed to go deeper into the research content to enhance prediabetes research.

## 5. Conclusion

The current study examined the research hotspots, frontiers, and development patterns in the field of prediabetes, with a focus on global research outcomes. The number of articles on prediabetes has increased over the last few decades, indicating that this new topic is gaining traction. Our findings provide an overview of the current status of diabetes research and have significant implications for future research directions.

## Data availability statement

The original contributions presented in the study are included in the article/Supplementary material, further inquiries can be directed to the corresponding author.

## Author contributions

JZ wrote the first draft of the editorial. ML reviewed and provided feedback on the manuscript. Both authors contributed to the article and approved the submitted version.

## Footnotes

^1^ https://www.science.org/content/article/war-prediabetes-could-be-boon-pharma-it-good-medicine

^2^ https://www.indexmundi.com/facts/china/research-and-development-expenditure
