# Supplementary figures and images for: Worldwide trends in prediabetes from 1985 to 2022: A bibliometric analysis using bibliometrix R-tool
Source: Front Public Health. 2023 Feb 13;11:1072521. doi: 10.3389/fpubh.2023.1072521 (PMC9993478; doi:10.3389/fpubh.2023.1072521)

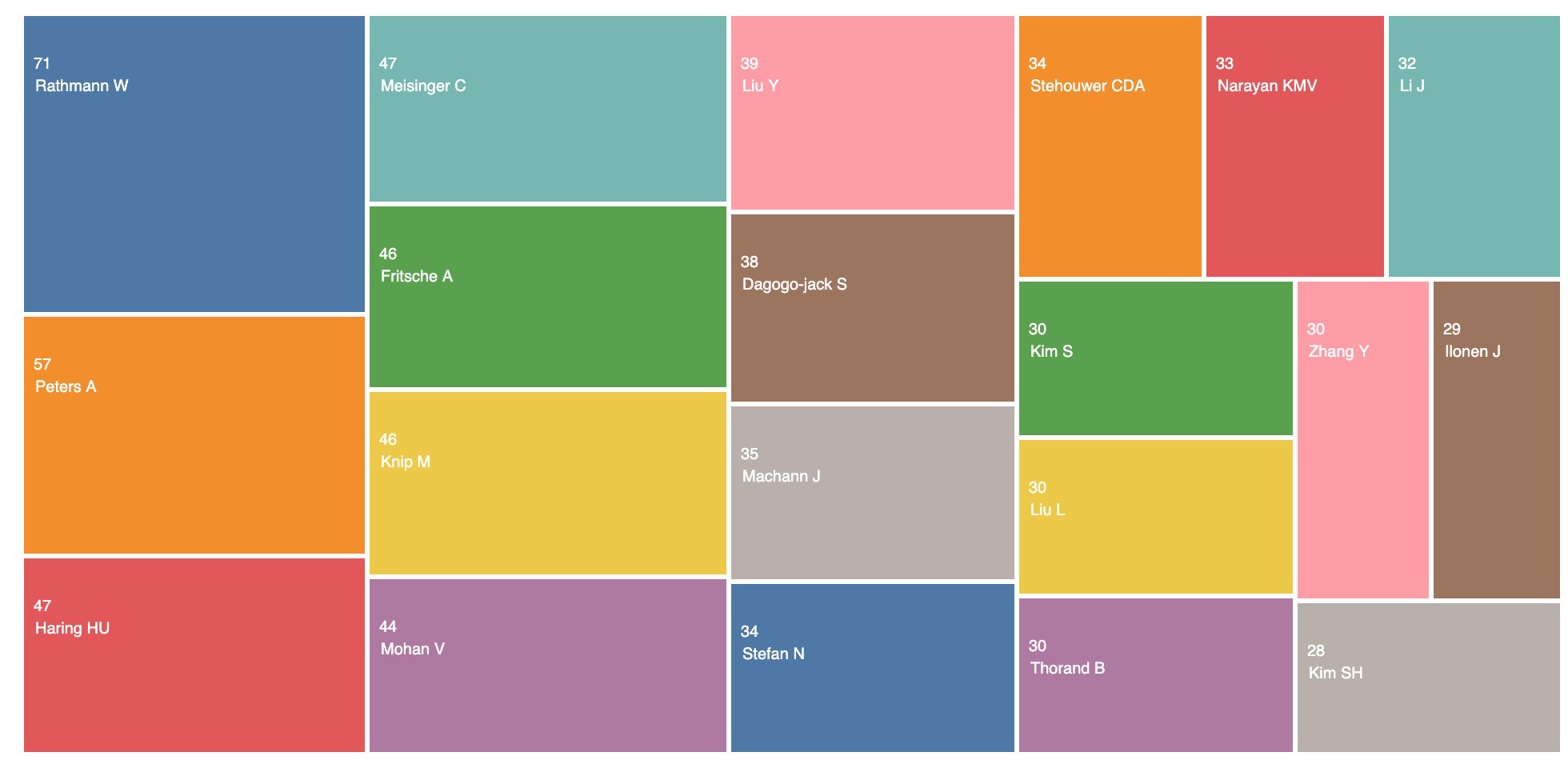

Supplement: Supplementary file 2 [file Image_1.JPEG]

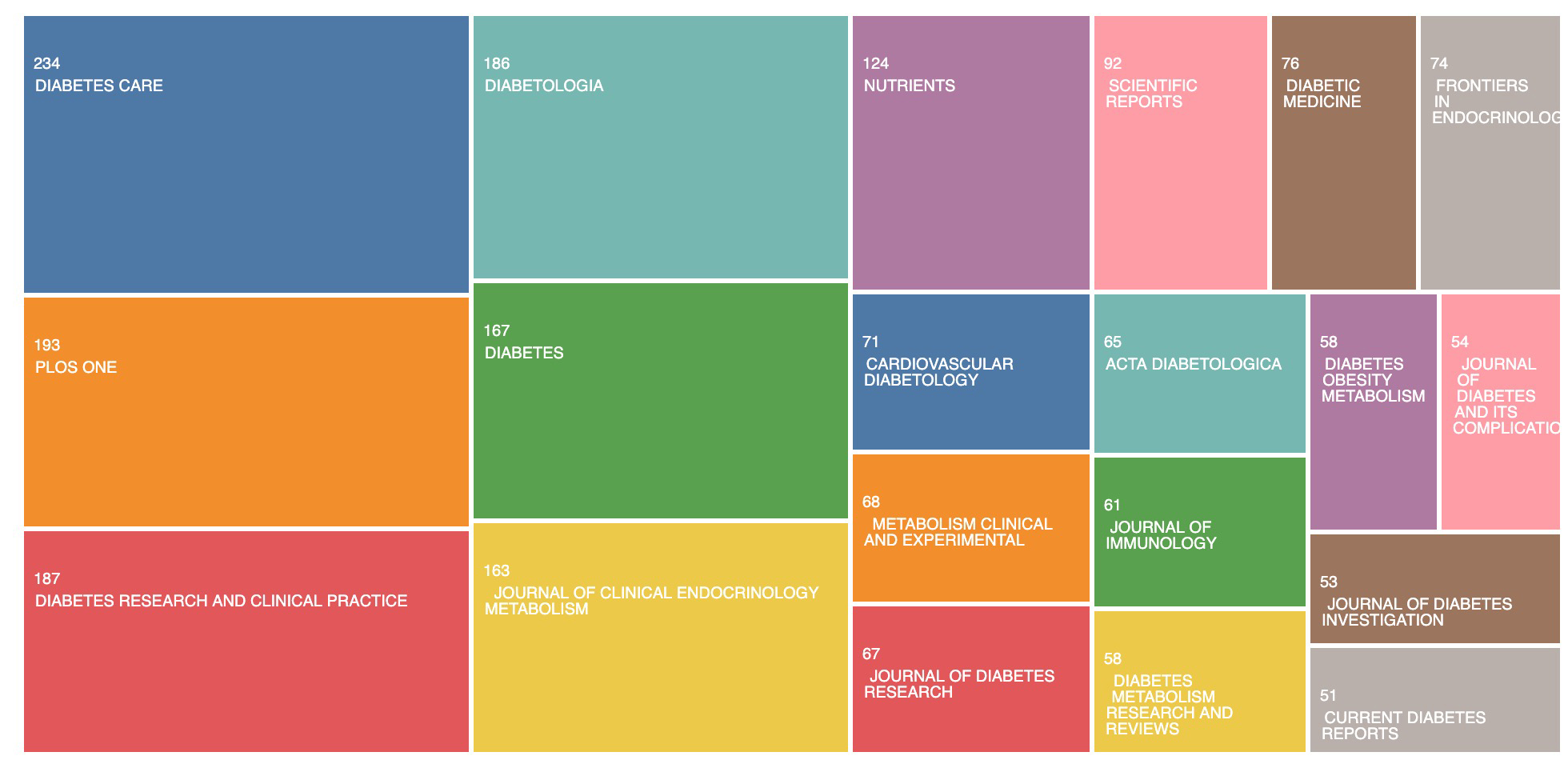

Supplement: Supplementary file 3 [file Image_2.JPEG]

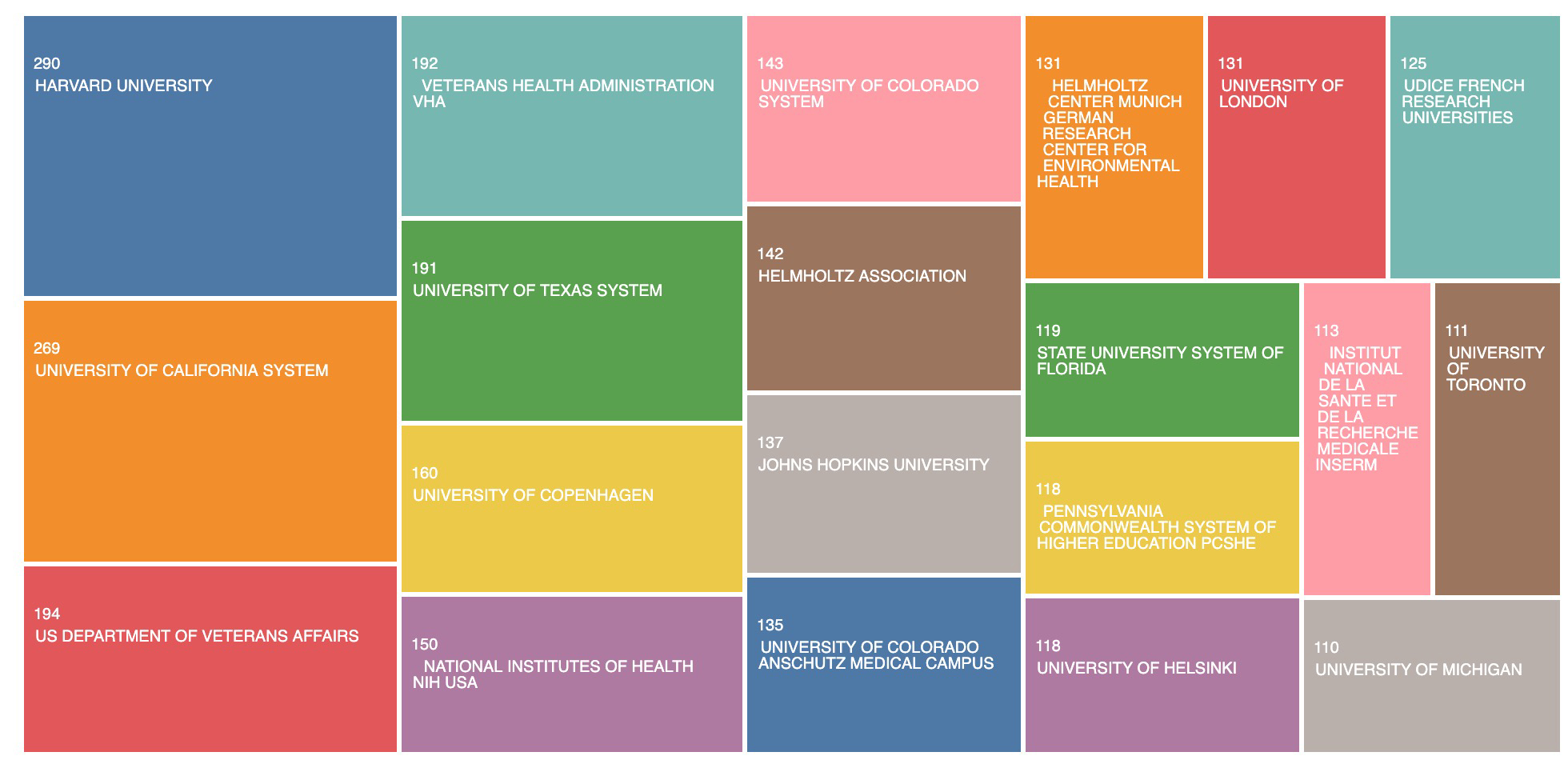

Supplement: Supplementary file 4 [file Image_3.JPEG]

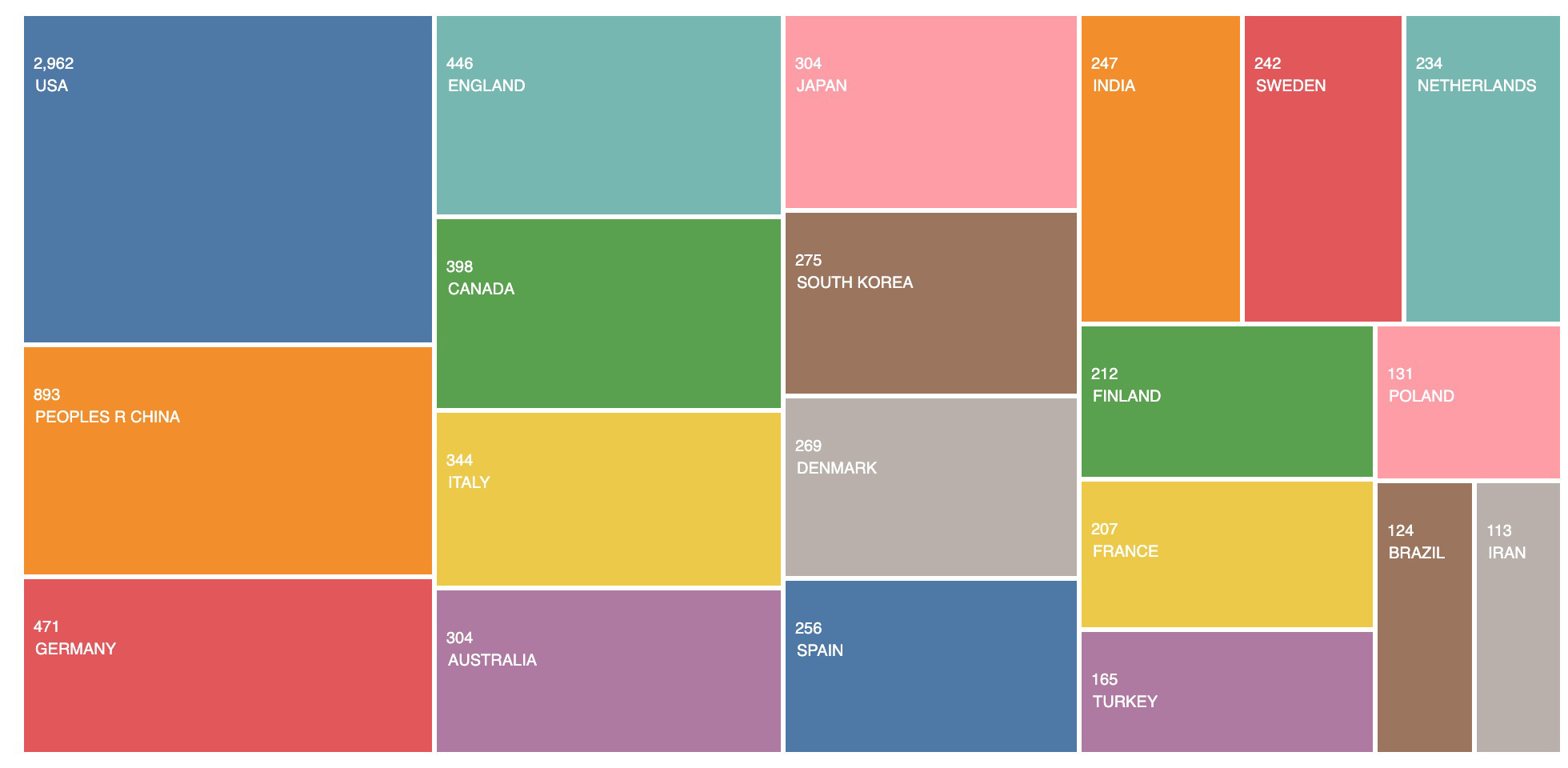

Supplement: Supplementary file 5 [file Image_4.JPEG]
